# Downregulation of LKB1/AMPK Signaling in Blood Mononuclear Cells Is Associated with the Severity of Guillain–Barre Syndrome

**DOI:** 10.3390/cells11182897

**Published:** 2022-09-16

**Authors:** Verica Paunovic, Stojan Peric, Irena Vukovic, Marina Stamenkovic, Emina Milosevic, Danijela Stevanovic, Milos Mandic, Ivana Basta, Ivana Berisavac, Mirjana Arsenijevic, Ivo Bozovic, Marko Nikolic, Zorica Stevic, Vladimir Trajkovic

**Affiliations:** 1 Institute of Microbiology and Immunology, Faculty of Medicine, University of Belgrade, Dr. Subotica 1, 11000 Belgrade, Serbia; 2 Neurology Clinic, University Clinical Center of Serbia, Faculty of Medicine, University of Belgrade, Dr. Subotica 6, 11000 Belgrade, Serbia

**Keywords:** Guillain–Barré syndrome, peripheral blood mononuclear cells, AMP-activated protein kinase, autophagy, mTOR, metformin

## Abstract

AMP-activated protein kinase (AMPK) is an intracellular energy sensor that regulates metabolic and immune functions mainly through the inhibition of the mechanistic target of rapamycin (mTOR)-dependent anabolic pathways and the activation of catabolic processes such as autophagy. The AMPK/mTOR signaling pathway and autophagy markers were analyzed by immunoblotting in blood mononuclear cells of 20 healthy control subjects and 23 patients with an acute demyelinating form of Guillain–Barré syndrome (GBS). The activation of the liver kinase B1 (LKB1)/AMPK/Raptor signaling axis was significantly reduced in GBS compared to control subjects. In contrast, the phosphorylated forms of mTOR activator AKT and mTOR substrate 4EBP1, as well as the levels of autophagy markers LC3-II, beclin-1, ATG5, p62/sequestosome 1, and NBR1 were similar between the two groups. The downregulation of LKB1/AMPK signaling, but not the activation status of the AKT/mTOR/4EBP1 pathway or the levels of autophagy markers, correlated with higher clinical activity and worse outcomes of GBS. A retrospective study in a diabetic cohort of GBS patients demonstrated that treatment with AMPK activator metformin was associated with milder GBS compared to insulin/sulphonylurea therapy. In conclusion, the impairment of the LKB1/AMPK pathway might contribute to the development/progression of GBS, thus representing a potential therapeutic target in this immune-mediated peripheral polyneuropathy.

## 1. Introduction

Guillain–Barré syndrome (GBS) is a heterogeneous acute autoimmune polyradiculoneuropathy with still ill-defined etiology. Although fairly rare (1–2 cases per 100,000 individuals), GBS is a considerable socioeconomic burden, since it may cause long-term disability and high mortality despite hospitalization and expensive therapy with intravenous immunoglobulins (IVIG) and/or plasma exchange [1,2,3]. The most common forms of GBS with distinct clinical presentations are acute inflammatory demyelinating polyneuropathy (AIDP), acute motor axonal neuropathy (AMAN), and acute motor-sensory axonal neuropathy (AMSAN) [4]. The pathogenesis of GBS includes the loss of immunological tolerance to myelin (AIDP) or axonal (AMAN/AMSAN) components in both T and B cell compartments, usually triggered by a gastrointestinal tract or respiratory infection and leading to inflammatory damage of peripheral nerves [5,6]. However, the molecular mechanisms underlying the initiation and progression of this pathogenic immune response are still poorly understood.

Immune cells rely on the precise regulation of intracellular metabolic pathways for their activation, proliferation, function, and survival [7,8]. The intracellular energy sensor AMP-activated protein kinase (AMPK) and the mechanistic target of rapamycin (mTOR), the master metabolic regulator, control immune cell differentiation and effector functions through the modulation of lineage-specific gene transcription and cytokine production [9]. Moreover, the AMPK/mTOR signaling axis regulates macroautophagy (hereafter autophagy), the fundamental mechanism for the autodigestion/recycling of cytoplasmic macromolecules and damaged organelles, which are sequestered in autophagic vesicles—autophagosomes, and delivered to lysosomes for degradation [10]. Autophagy is regulated both transcriptionally, by modulating the expression of autophagy-related (ATG) genes, and by post-translational modifications and controlled sequential interaction of their protein products [11]. The main autophagy checkpoints include beclin-1 (mammalian ATG6)-dependent initiation of autophagosome biogenesis, ATG5-dependent lipidation of ATG8 leading to autophagosome formation and expansion, and the loading of autophagosomes with ubiquitinated intracellular cargo delivered by autophagy receptors p62/sequestosome 1 (SQSTM1) and neighbor of BRCA1 gene 1 (NBR1) [10,11]. Autophagosomes then fuse with lysosomes, where cellular components, including SQSTM1 and NBR1, are eventually degraded [10,11]. The principal autophagy regulator that integrates growth factor and nutrient signals is mTOR-containing mTOR complex 1 (mTORC1), which blocks autophagy initiation through the phosphorylation of Unc-51-like autophagy-activating kinase 1 (ULK1, mammalian ATG1) [12]. Growth factor-stimulated kinase AKT inhibits autophagy via mTORC1 activation, while liver kinase B1 (LKB1)-activated AMPK induces autophagy by the direct activation of ULK1 and/or inhibition of mTORC1 through the phosphorylation of tuberous sclerosis complex 2 or regulatory-associated protein of mTOR (Raptor) [12]. The extracellular signal-regulated kinase (ERK), p38 mitogen-activated protein kinase (MAPK), and protein kinase C (PKC) have also been reported to modulate autophagy through both mTORC-1-dependent and -independent mechanisms [13,14,15,16].

Besides serving as a homeostatic/quality control mechanism and providing energy and building blocks for cellular maintenance in stressful conditions, autophagy has been implicated in the regulation of the immune response. Immunological signals modulate autophagy, which in turn regulates innate and adaptive immune responses by influencing inflammasome activity, antigen processing/presentation, and T and B cell development, activation, and function [17]. Accordingly, autophagy modulation is involved in animal models of autoimmunity, and genome-wide association studies show the association between ATG gene polymorphisms and several autoimmune/inflammatory disorders [18]. While autophagy alterations have been demonstrated in the peripheral nerves of rats with chronic experimental autoimmune neuritis [19], the activation status of autophagy, AMPK/mTORC1, and other autophagy-related signaling pathways in human inflammatory neuropathies, including GBS, has not been explored so far.

The present study aimed to examine the regulation of AMPK/mTORC1, autophagy, and related intracellular signaling pathways (AKT, ERK, p38 MAPK, PKC) in peripheral blood mononuclear cells (PBMCs) of GBS patients. Our results indicate that the inhibition of the leukocyte LKB1/AMPK signaling axis might be associated with the clinical severity of GBS independently of autophagy, mTORC1, and other autophagy-related signaling pathways. Accordingly, treatment with AMPK activator metformin, compared to insulin/sulphonylurea therapy, was associated with milder GBS symptoms in patients with type 2 diabetes mellitus.

## 2. Materials and Methods

### 2.1. Study Subjects and PBMCs Isolation

For the analysis of autophagy and related signaling pathways, blood samples from 23 GBS patients with AIDP were collected upon their admission to the Neurology Clinic, Clinical Center of Serbia (Belgrade, Serbia) between 1 January 2019, and 31 December 2019. Blood samples of 20 sex/age-matched healthy control subjects were collected from healthy employees of the Clinic and their relatives. All patients included in the study fulfilled the Brighton criteria for GBS [20] and were drug-naïve at the time of sampling. The patients with axonal subtypes of GBS (AMAN/AMSAN), which are less frequent in Europe and North America [4], as well as the patients with rare GBS variants such as Miller Fisher syndrome and pharyngocervical brachial variants, were excluded from the analysis to obtain a more homogenous study group. The patients and control subjects with other systemic acute/chronic diseases or those receiving any therapy that may affect their sensory/motor function or autophagy [21] were also excluded from the study. At the time of sampling, the GBS patients’ disability was evaluated using the GBS disability scale (GDS; 0–6 ranging from no disability to death) [22], wherein a score >1 indicated poor recovery on follow-up after 6 months (two patients refused control examination). Motor impairment at diagnosis was scored using the Medical Research Council sum score (MRCSS; range 0–60, with 0 denoting complete paralysis of all 12 analyzed muscles and 60 points denoting preserved muscle strength in all muscles) [23]. Blood samples (40 mL) were collected by venipuncture into anticoagulant (ethylenediaminetetraacetic acid)-containing tubes (BD, Franklin Lakes, NJ, USA). PBMCs were isolated using Lymphoprep (Axis-Shield, Oslo, Norway) gradient according to the manufacturer’s instructions. The study was approved by the Ethics Committee of the Faculty of Medicine, University of Belgrade, and all subjects gave written consent to participate.

### 2.2. Retrospective Study of Metformin Influence on GBS

To analyze the effect of metformin therapy on GBS, we used the national Registry of Rare Neuromuscular Diseases to retrieve the clinical data of 49 GBS patients with preexisting type 2 diabetes mellitus and no other acute/chronic illnesses, treated for GBS at the Neurology Clinic, University Clinical Center of Serbia (Belgrade, Serbia) in ten years from 1 January 2009 to 1 January 2019 The patients were treated for diabetes with metformin, insulin, or sulphonylurea derivatives (gliclazide, glimepiride, or glibenclamide). The severity of GBS at nadir and hospital discharge was defined as mild (GDS ≤ 3) or high (GDS ≥ 4). The Registry of Rare Neuromuscular Diseases was approved by the Ethics Committee of the Faculty of Medicine, University of Belgrade (Belgrade, Serbia), while the Institutional Review Board approved the protocol of the study.

### 2.3. RT-qPCR Analysis

RNA was extracted from PBMCs using RNeasy Protect Mini Kit (Qiagen, Hilden, Germany) and reverse-transcribed with MuLV reverse transcriptase and random hexamers (Thermo Fisher Scientific, Waltham, MA, USA), following the manufacturer’s recommendations. RTqPCR was performed in a Realplex2 Mastercycler (Eppendorf, Hamburg, Germany) using MicroAmp^®^ Optical 96-well reaction plates, TaqMan Universal PCR Master Mix, and TaqMan primers/probes (all from Thermo Fisher Scientific, Waltham, MA, USA) for human activating transcription factor 4 (*ATF4*; Hs00909569_g1), *ATG3* (Hs00223937_m1), *ATG4B* (Hs00367088_m1), *ATG7* (Hs00197348_m1), *ATG10* (Hs009197718_m1), *ATG13* (Hs00207186_m1), *ATG14* (Hs00208732_m1), B-cell lymphoma 2 interacting protein 3 (*BNIP3*; Hs00969291_m1), forkhead box O1 (*FOXO1*; Hs01054576_m1), *FOXO3* (Hs00921424_m1), beclin-1 (*BECN1*; Hs00186838_m1), γ-aminobutyric acid receptor-associated protein (*GABARAP*; Hs00925899_g1), *SQSTM1* (Hs00177654_m1), microtubule-associated protein 1 light chain 3B (*LC3B*; Hs01076567_g1), *ULK1* (Hs00177504_m1), phosphatidylinositol 3-kinase catalytic subunit type 3 (*PIK3C3*; Hs00176908_m1), transcription factor EB (*TFEB*; Hs00292981_m1), and hypoxanthine phosphoribosyltransferase 1 (*HPRT1*; Hs02800695_m1) and TATA box binding protein (*TBP*; Hs99999910_m1) as housekeeping genes. The stability of *HPRT1* and *TBP* mRNA levels across samples was confirmed by BestKeeper [24] and NormFinder [25] algorithms (data not shown). The assays were performed in duplicates following the manufacturer’s instructions. The geomean cycle of threshold (Ct) values of *HPRT1*/*TBP* genes was subtracted from the Ct values of target genes to obtain dCt as a measure of the target mRNA level.

### 2.4. Immunoblot Analysis

PBMCs were lysed in RIPA buffer (150 mM NaCl, 1.0% IGEPAL^®^ CA-630, 0.5% sodium deoxycholate, 0.1% SDS, 50 mM Tris, pH 8.0) containing protease/phosphatase inhibitor cocktail (Merck, Burlington, MA, USA). Cell lysates were incubated on ice for 30 min, centrifuged at 14,000× *g* for 15 min at 4 °C, and the supernatants were collected. Equal protein amounts from each sample were separated by SDS-PAGE electrophoresis and transferred to a nitrocellulose membrane (Amersham Protran; GE Healthcare, Little Chalfont, UK). After blocking with 10% milk powder, membranes were incubated with primary rabbit antibodies against SQSTM1 (NBP1-48320; Novus Biologicals, Littleton, CO, USA), NBR1 (#9891), LC3B (#2775), beclin-1 (#3495), ATG5 (#12994), LKB1 (#3047), phospho-LKB1 (Ser428; #3482), AMPKα (#2603), phospho-AMPKα (Thr172; #2535), Raptor (#2280), phospho-Raptor (Ser792; #2083), phospho-eukaryotic translation initiation factor 4E-binding protein 1 (4EBP1) (Thr37/46; #2855), phospho-AKT (Ser473; #9271), phospho-ERK1/2 (Thr202/Tyr204; #9101), phospho-p38 MAPK (Thr180/Tyr182; #9211), phospho-glycogen synthase kinase 3β (GSK3β, Ser9; #9322), and actin (#4968) as a loading control (all from Cell Signaling Technology, Cambridge, MA, USA). The peroxidase-conjugated goat anti-rabbit IgG (111-035-144) and anti-mouse IgG2a (115-035-206) (both from Jackson ImmunoResearch, West Grove, PA, USA) were used as secondary antibodies. The protein bands were visualized by enhanced chemiluminescence using the ChemiDoc imaging system (Bio-Rad, Hercules, CA, USA), and densitometric quantification was performed using ImageLab software (Bio-Rad, Hercules, CA, USA). For the measurement of total amounts of LKB1, AMPK, and Raptor, the membranes were stripped after detecting the phosphorylated proteins (the efficiency of stripping was confirmed by incubating the membrane with a chemiluminescent detection reagent), and the membranes were washed, blocked, and re-probed with appropriate antibodies. Actin was detected on the same membrane simultaneously with the specific protein or after stripping, depending on the molecular weight of the latter. The absolute levels of phosphorylated and total proteins were calculated relative to actin, while the relative activation of specific signaling molecules was assessed as the ratio of phosphorylated and total protein levels. An internal calibrator was used for the normalization of samples across different gels. The original blots of phosphorylated and/or total proteins with corresponding actin blots are shown in Supplementary Figures S1 (AMPK, LKB1), S2 (Raptor, LC3I/II), S3 (ATG5, beclin-1, SQSTM1), S4 (4EBP1, ERK, GSK3β), S5 (AKT, p38 MAPK), and S6 (NBR1).

### 2.5. Statistical Analysis

Because most of the patients’ and control subjects’ data failed to meet normality, homogeneity of variance, and/or “no outliers” assumptions, statistical analysis was performed using nonparametric tests. The two-tailed Mann–Whitney U test was used for inter-group comparisons, while Spearman’s rank-order test was used to assess correlations (the linear trendlines in correlation graphs are only for visualization of correlation trends, and do not reflect the exact relationship between variables). The frequencies were compared with Fisher’s exact test. The binary logistic regression (enter method) was employed for multivariate analysis after excluding multicollinearity (variance inflation factor < 3), while the Box–Tidwell test was used to confirm the linearity between the independent predictors and the logit transformation of the dependent variable. The goodness of fit was assessed by the Hosmer–Lemeshow test, *p* > 0.05 indicating a good fit. The statistical analysis was performed using IBM SPSS software (v22), and the significance level was set to *p* ≤ 0.05. To reduce the risk of missing the true effects, no corrections for multiple comparisons were made [26].

## 3. Results

### 3.1. Demographic and Clinical Characteristics of Study Subjects

The GBS and control groups did not significantly differ in sex distribution (male/female 14/9 vs. 11/9, *p* = 0.763, Fisher’s exact test) or age (median/interquartile range 59/46–69 vs. 59/48–69 years, *p* = 0.817, Mann–Whitney U test). The sex distribution in the GBS group was consistent with the fact that men are more frequently affected than women (ratio 3:2) [1]. The clinical features of GBS patients, including GDS as a measure of overall disability, MRCSS as a measure of muscle strength, the presence of gastrointestinal/respiratory infection as a precipitating factor, mode of therapy, and death rate are presented in Table 1. All patients were treated with IVIG, and the death rate of 13% was consistent with the reported GBS mortality rates in Serbia [3]. Neither of the two disease severity measures, GDS (at admission and 6 months) or MRCSS at admission, were significantly associated with the presence of gastrointestinal/respiratory infection before disease onset (Supplementary Figure S7). While GDS values did not significantly differ between male and female patients, the MRCSS values were slightly but significantly higher in males (Supplementary Figure S7). Moreover, there was a significant positive correlation between patients’ age and GDS at admission, indicating an increase in disease severity with age (Supplementary Figure S7). No correlation was observed between patients’ age and MRCSS values (Supplementary Figure S7).

### 3.2. AMPK Signaling Pathway Is Downregulated in PBMCs of GBS Patients

**Figure 1:**
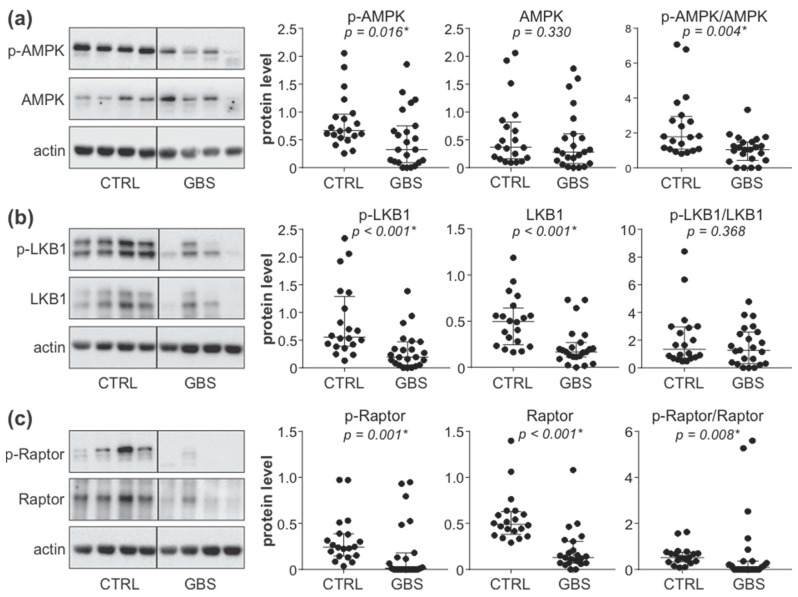
Downregulation of AMPK signaling in the PBMCs of GBS patients. (**a**–**c**) PBMCs were isolated from control subjects (CTRL; *n* = 20) and GBS patients (*n* = 23), and the levels of phosphorylated and total forms of AMPK (**a**), LKB1 (**b**), and Raptor (**c**) were analyzed by immunoblotting. The representative blots are shown, while densitometry results are presented relative to actin or as phospho/total signal ratio (horizontal lines represent median and interquartile range; * *p* < 0.05, two-tailed Mann–Whitney U test).

To analyze the status of the AMPK signaling axis in the PBMCs of control and GBS subjects, we measured the expression and phosphorylation of AMPK, its upstream activator LKB1, and AMPK substrate Raptor. The results of immunoblot analysis revealed that the levels of phosphorylated AMPK (Figure 1a), phosphorylated and total LKB1 (Figure 1b), as well as phosphorylated and total Raptor (Figure 1c), were significantly lower in the PBMCs of GBS patients compared to control subjects. The ratio of phosphorylated and total LKB1 levels was not significantly altered in GBS PBMCs (Figure 1b), indicating that the decrease in phospho-LKB1 was mainly a consequence of reduced LKB1 expression. On the other hand, the phospho/total ratio of both AMPK (Figure 1a) and Raptor (Figure 1c) was significantly reduced in GBS patients, thus confirming that the phosphorylation of AMPK and Raptor was impaired in GBS leukocytes. This was expected having in mind that in the AMPK signaling cascade, LKB1 phosphorylates AMPK, which then phosphorylates Raptor. These data demonstrate that the activity of the LKB1/AMPK/Raptor signaling axis in the PBMCs of GBS patients is downregulated at several levels.

### 3.3. Autophagy Markers and AKT/mTORC1 Pathway Are Not Affected in PBMCs of GBS Patients

**Figure 2:**
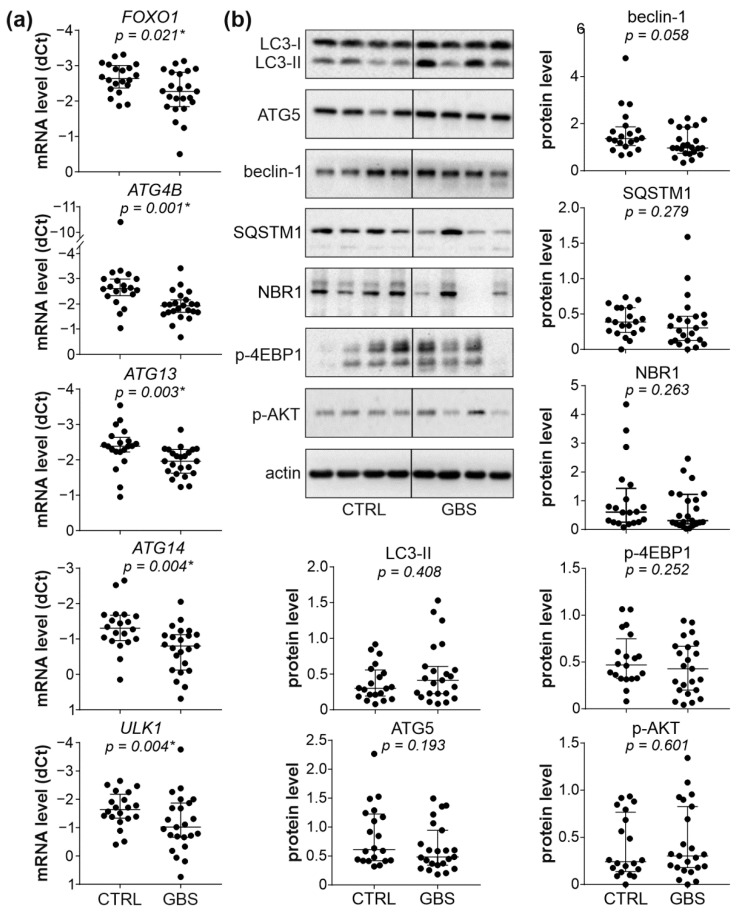
Autophagy and AKT/mTORC1 signaling in PBMCs of GBS patients. (**a**,**b**) PBMCs were isolated from control subjects (CTRL; *n* = 20) and GBS patients (*n* = 23). (**a**) The mRNA levels of FOXO1, ATG4B, ATG13, ATG14, and ULK1 were determined by RT-qPCR and expressed as dCT values (lower dCT values correspond to higher expression). (**b**) The protein levels of LC3-I/II, ATG5, beclin-1, SQSTM1, NBR1, phospho-AKT, and phospho-4EBP1 were analyzed by immunoblotting, and the representative blots are shown together with densitometry data (relative to actin). (**a**,**b**) The dCT and densitometry values of each sample are presented, with horizontal lines representing median values and interquartile range (* *p* < 0.05, two-tailed Mann–Whitney U test).

We next assessed the status of autophagy in the PBMCs of GBS patients by examining the expression of autophagy genes, the protein levels of pro-autophagic regulators beclin-1 and ATG5, the conversion of LC3-I (a mammalian homolog of ATG8) to autophagosome associated LC3-II, and autophagy-selective degradation of cargo receptors SQSTM1 and NBR1. The RT-qPCR analysis revealed that out of seventeen autophagy regulators tested, the levels of mRNA encoding autophagy transcription factor FOXO1 and four ATG molecules (ATG4B, ATG13, ATG14, and ULK1, a mammalian homolog of ATG1), were significantly reduced in the PBMCs of GBS patients compared to control subjects (Figure 2a and Supplementary Figure S8). However, the immunoblot analysis demonstrated that the protein levels of autophagy markers LC3-II, ATG5, beclin-1, SQSTM1, and NBR1 did not significantly differ between the PBMCs of GBS patients and healthy controls (Figure 2b). Similarly, the activation of the autophagy-regulating AKT/mTORC1 signaling axis, assessed by measuring the phosphorylation of AKT and mTORC1 substrate 4EBP1, was not altered in GBS leukocytes (Figure 2b). Therefore, despite the partial suppression of ATG transcription, the expression of autophagy markers and the activity of the AKT/mTORC1 pathway were mainly unaffected in GBS leukocytes.

### 3.4. Interplay between AMPK, ERK, and PKC Signaling Pathways in PBMCs of GBS Patients

**Figure 3:**
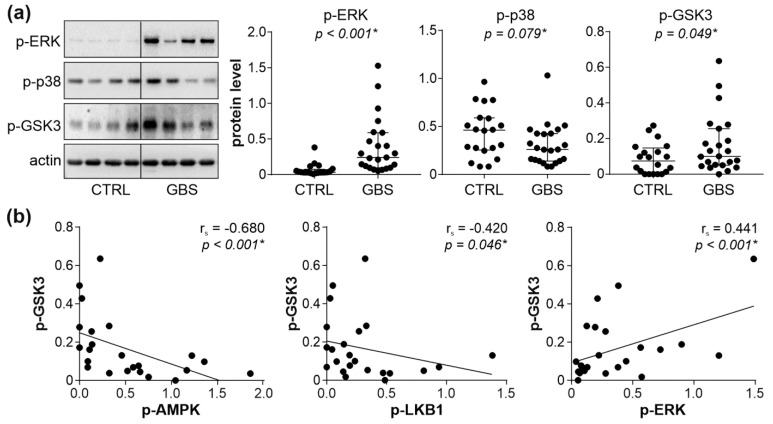
AMPK–ERK–PKC interplay in GBS PBMCs. (**a**,**b**) PBMCs were isolated from control subjects (CTRL; *n* = 20) and GBS patients (*n* = 23), and the levels of phosphorylated ERK, p38 MAPK, and GSK3β were assessed by immunoblotting. The representative blots and densitometry data (relative to actin) are shown in (**a**), with horizontal lines representing median values and interquartile range (* *p* < 0.05, two-tailed Mann–Whitney U test). The correlations between GSK3β and phospho-AMPK, phospho-LKB1, or phospho-ERK are presented in (**b**) (r_s_—Spearman’s correlation coefficient; * *p* < 0.05, Spearman’s rank-order test).

To account for the apparent absence of autophagy suppression in the face of AMPK downregulation in GBS PBMCs, we assessed the activation of other autophagy-activating signaling pathways, namely ERK, p38 MAPK, and PKC. The phosphorylation of ERK and PKC substrate GSK3β, but not p38 MAPK, was significantly increased in the PBMCs of GBS patients compared to control subjects (Figure 3a). We next assessed if the observed increase in PKC and/or ERK activation might be connected to AMPK downregulation in the PBMCs of GBS patients. The phosphorylation of AMPK and LKB1, as well as the total levels of LKB1, were inversely correlated with those of phosphorylated GSK3β, while a positive correlation was observed between the phosphorylated forms of ERK and GSK3β (Figure 3b). No significant correlation was found between the phosphorylation of p38 MAPK and GSK3β, nor between the levels of phospho-ERK or phospho-p38 and phospho-AMPK, phospho-LKB1, or total LKB1 levels (Supplementary Figure S9). Collectively, these data indicate that the activation of ERK and PKC might counterbalance AMPK inhibition-mediated autophagy suppression in GBS leukocytes, with PKC possibly being connected with both AMPK downregulation and ERK activation.

### 3.5. Downregulation of LKB1/AMPK Pathway Correlates with GBS Severity and Poor Outcome

**Figure 4:**
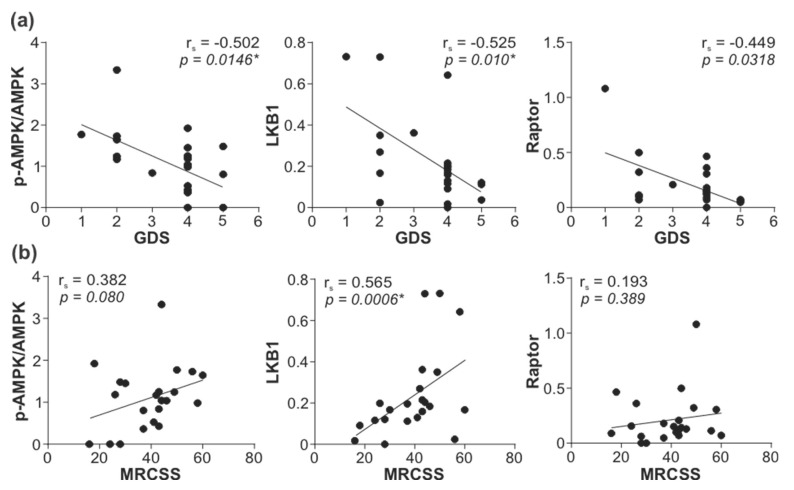
Correlation between leukocyte AMPK signaling and GBS severity. The levels of total LKB1, phospho/total AMPK ratio, and total Raptor in PBMCs of GBS patients were determined by immunoblotting, and the correlations with (**a**) GDS (*n* = 23) and (**b**) MRCSS (*n* = 22) were assessed (r_s_—Spearman’s correlation coefficient; * *p* < 0.05, Spearman’s rank-order test).

**Figure 5:**
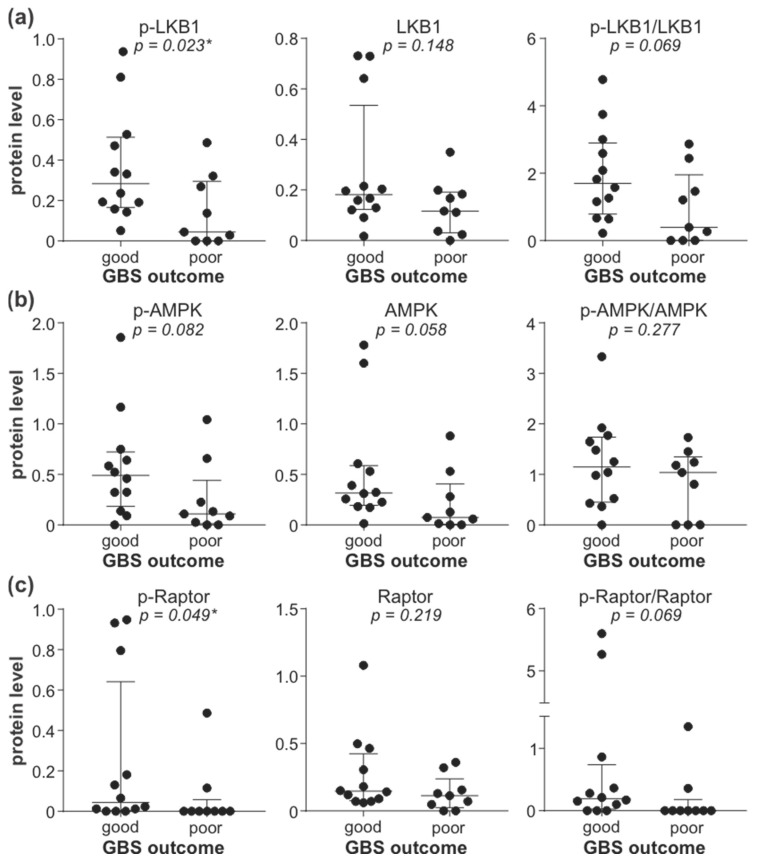
Association of leukocyte AMPK signaling with GBS outcome. The levels of phosphorylated, total, and phospho/total ratio of LKB1 (**a**), AMPK (**b**), and Raptor (**c**) in PBMCs of GBS patients (*n* = 21) were determined by immunoblotting and compared between patients with good (GDS ≤ 1, *n* = 12) and poor (GDS > 1, *n* = 9) disease outcome at 6 months (horizontal lines represent median and interquartile range; * *p* < 0.05, two-tailed Mann–Whitney U test).

To explore the possible role of reduced leukocyte AMPK signaling in GBS, we correlated the activity of the AMPK signaling axis with GDS and MRCSS as the measures of GBS patients’ disability and muscle strength. The total levels of LKB1, phospho/total AMPK ratio, and total levels of Raptor displayed a significant inverse correlation with GDS at hospital admission (Figure 4a). The total levels of LKB1 were also positively correlated with MRCSS values at hospital admission, while a trend toward a positive association of the phospho/total AMPK ratio and total levels of Raptor with MRCSS failed to reach statistical significance (Figure 4b). The observed correlations were not due to the confounding effect of age or sex, as they did not influence the expression/activation of AMPK signaling molecules associated with disease severity (total LKB1, phospho/total AMPK, total Raptor) (Supplementary Figure S10). The levels of phospho-LKB1, phospho-AMPK, total AMPK, and phospho-Raptor, as well as phospho/total LKB1 and phospho/total Raptor ratio, were not significantly correlated with either GDS or MRCSS, although a tendency toward negative correlation with GDS was evident in the case of phospho-Raptor and phospho/total Raptor ratio (Supplementary Figure S11). The lower levels of phospho-LKB1 (Figure 5a) and phospho-Raptor (Figure 5c) at admission were significantly associated with poor recovery (GDS > 1) at 6 months (Figure 5). A similar trend was observed with the phospho/total LKB1 ratio (Figure 5a), phospho-AMPK, and total AMPK levels (Figure 5b), and phospho/total Raptor ratio (Figure 5c), but the associations did not reach statistical significance. On the other hand, no significant associations were observed between GDS (at admission or after six months) and autophagy markers (LC3-II, ATG5, beclin-1, SQSTM1, and NBR1) or phosphorylated forms of 4EBP1, AKT, ERK, p38 MAPK, and GSK3β (Supplementary Figures S12 and S13). Therefore, the downregulation of the leukocyte AMPK signaling axis correlated with increased severity and poor outcome of GBS independently of age, sex, and the expression of autophagy markers or the activation of autophagy-related signaling pathways.

### 3.6. Treatment with Metformin Is Associated with Better GBS Outcome in Diabetic Patients

Finally, to assess the potential therapeutic benefit of counteracting AMPK signaling deficit in GBS, we compared the disease severity in GBS patients with type 2 diabetes treated with AMPK activator metformin and those receiving insulin and/or sulphonylurea derivatives without metformin. The two groups did not significantly differ in sex distribution, age, fasting blood glucose level at the time of admission, the presence of respiratory or gastrointestinal infection before GBS onset, or the proportion of patients who received IVIG/plasma exchange therapy (Table 2). On the other hand, the proportion of patients with mild disability (GDS ≤ 3) at disease nadir and hospital discharge was significantly higher in the metformin group (Table 2). To examine the prognostic value of metformin therapy in diabetic GBS patients, a simple binary logistic regression model for predicting GBS outcome at hospital discharge was created, incorporating patients’ age, preceding gastrointestinal infection, and the mode of diabetes treatment as independent variables. The model was significant (chi-square = 16.203, *p* = 0.001), explaining 38.9% (Nagelkerke R^2^ = 0.389) of the variance in GBS outcome, correctly classifying 75.5% of cases, and presenting a good fit to the data (Hosmer–Lemeshow *p* = 0.689). In addition to the absence of antecedent gastrointestinal infection, metformin therapy was a significant independent predictor of good recovery, with metformin-treated patients being almost six times more likely to have a favorable outcome (Table 3). The inclusion of patients’ sex and glycemia at admission, as a measure of diabetes control, did not further improve the performance of the model (Nagelkerke R^2^ = 0.399, correct prediction 75.5%) (Supplementary Table S1). These data indicate that diabetic patients receiving metformin are expected to experience milder GBS.

## 4. Discussion

To the best of our knowledge, the presented results suggest for the first time that the downregulation of leukocyte AMPK signaling might be associated with the severity/outcome of the AIDP form of GBS. Moreover, the possible therapeutic benefit of counteracting AMPK suppression in GBS is indicated by a retrospective analysis showing a milder disease in diabetic GBS patients treated with the AMPK activator metformin.

The main finding of the present study is that the activity of the LKB1/AMPK/Raptor signaling axis was significantly reduced in the PBMCs of AIDP patients compared to age/sex-matched healthy control subjects. The initial defect was presumably the decrease in the expression of LKB1, which subsequently caused the suppression of the downstream signaling molecules AMPK and Raptor. However, it is also possible that AMPK and/or Raptor were downregulated at least in part independently of LKB1, as actually indicated by the decrease in the total levels of Raptor in AIDP leukocytes. Similar to our results, AMPK downregulation in T cells and peripheral blood lymphocytes has previously been reported in a mouse model of multiple sclerosis and human hereditary metabolic/neuroinflammatory disease X-linked adrenoleukodystrophy, respectively [27,28]. These previous studies and our data support the possible involvement of dysregulated leukocyte AMPK signaling in immune-mediated central and peripheral demyelinating disorders. Indeed, AMPK has been increasingly recognized as an important regulator of the metabolism, maintenance, expansion, and function of both T and B cell lineages [29,30,31]. While the exact mechanisms of AMPK involvement in the neuroinflammatory damage in AIDP remain to be investigated, its protective role has been suggested by an association between the downregulation of different AMPK signaling members and increased disability and/or motor impairment in GBS. Moreover, the lower activation of the LKB1/AMPK/Raptor pathway in peripheral blood leukocytes was associated with poor recovery from AIDP, indicating a potential prognostic value of leukocyte AMPK signaling status in this disease. However, this association, although significant, was rather weak, thus remaining to be confirmed in larger studies. Also, while the present study was limited to the analysis of the whole PBMC population, we plan to use cell sorting and immunocytochemistry to further evaluate AMPK signaling in specific PBMC subpopulations.

Having in mind the well-known role of AMPK in relieving autophagy from mTORC1 suppression [32], it is somewhat unexpected that the expression of autophagy markers and mTORC1 activity in the PBMCs of GBS patients remained unchanged. This is even more surprising given that AMPK downregulation in GBS leukocytes was associated with reduced mRNA levels of autophagy transcription factor FOXO1 and four ATG molecules involved in autophagy induction (ULK1 and ATG13), the initiation of autophagosome formation (ATG14), and the cleavage of LC3 to generate LC3-II precursor LC3-I (ATG4B) [10]. While this agrees with the involvement of AMPK in ATG transcription [33], the observed AMPK dissociation from mTORC1 signaling and expression of autophagy markers could stem from the counterbalancing activation of other signaling pathways known to modulate mTORC1/autophagy, such as ERK, PKC, or p38 MAPK [13,14,15,16]. Indeed, while p38 MAPK activation was not altered in GBS leukocytes, the phosphorylation of ERK and PKC substrate GSK3β, reflecting PKC activity, was significantly enhanced. Accordingly, the previous analysis of the transcriptional profile of GBS leukocytes revealed the upregulation of both ERK and PKC pathways [34]. Moreover, our findings that PKC activity was correlated inversely with AMPK and positively with ERK phosphorylation, indicate that PKC could contribute to both AMPK inhibition and ERK activation in GBS leukocytes. This is consistent with the ability of PKC to inhibit AMPK [35,36,37,38] and activate ERK [39,40,41,42] in various experimental settings. Despite the increased activity, neither PKC nor ERK was associated with GBS disability or motor impairment scores, thus further emphasizing the potential value of AMPK signaling status as a marker of GBS severity and outcome. AMPK phosphorylates a variety of metabolic targets [43], which might affect immune cells independently of mTORC1 and/or autophagy. However, it should be noted that we analyzed the steady-state levels of an autophagosome marker LC3-II and autophagy-selective targets SQSTM1 and NBR1 as a proxy for autophagic status, without directly evaluating autophagic turnover (flux). We are currently seeking to confirm the absence of autophagy modulation in GBS by using a recently developed protocol for measuring autophagic flux in whole blood, thus avoiding cell cultivation in nutrient-rich media that affect mTOR signaling and autophagy [44].

To assess the possible usefulness of AMPK as a therapeutic target in GBS, we exploited the fact that the anti-diabetic drug metformin achieves its insulin-sensitizing effect primarily through AMPK activation [45]. The milder GBS symptoms observed in metformin-treated, compared to insulin/sulphonylurea-treated GBS patients with type 2 diabetes, indicates that pharmacological reversal of AMPK signaling block might be associated with reduced GBS severity. Moreover, while extending to type 2 diabetes the previous findings on preceding diarrhea as a predictive factor of poor GBS outcome [46], we demonstrate that metformin treatment was a significant independent predictor of favorable GBS outcome at hospital discharge. While the effects of metformin in immune-mediated damage of peripheral nerves are yet to be explored, our data are consistent with its ability to reduce inflammatory CNS demyelination in animal models of multiple sclerosis via an AMPK-dependent mechanism [47,48]. However, we and others have previously shown that diabetes worsens the prognosis of GBS [49,50,51], possibly due to the presence of pre-existing nerve injury, reduced capacity for nerve regeneration, and/or systemic inflammation [52,53,54]. The non-metformin group in our study mainly included patients treated with insulin, a second-line medication for type 2 diabetes patients in which hyperglycemia could not be controlled with metformin [55]. Hence, it is possible that the observed association between metformin treatment and better GBS outcome was actually due to inherently milder diabetes in the metformin group. On the other hand, the absence of association between fasting glycemia at admission and metformin treatment or GBS outcome indicates that the last two variables might be connected independently of diabetes control. Nevertheless, more extensive studies are required to corroborate the proposed beneficial effect of metformin in GBS.

## 5. Conclusions

The present report indicates that the impairment of the AMPK pathway in PBMCs might contribute to the development and/or progression of the AIDP form of GBS, thus representing a possible therapeutic target in this autoimmune disorder. Accordingly, restoring the defective leukocyte AMPK signaling with AMPK-activating drugs, such as clinically approved antidiabetic metformin, might be worth considering as a novel add-on therapeutic strategy in GBS. Larger studies are required to confirm these observations and extend them to other forms of GBS and/or specific leukocyte subpopulations, as well as to validate the prognostic/therapeutic potential of assessing/manipulating the leukocyte AMPK signaling axis in this immune-mediated neuropathy.

## Figures and Tables

**Table 1:** Clinical characteristics of GBS patients.

Clinical Characteristics	Values
GDS	
–at admission (*n* = 23)	4 (2–4) ^a^
–at 6 months (*n* = 21) ^b^	1 (0.5–3) ^a^
MRCSS at admission (*n* = 22) ^c^	42.5 (28–47) ^a^
Previous infection	14 (60.8%)
–gastrointestinal	7 (30.4%)
–respiratory	7 (30.4%)
IVIG therapy	23 (100%)
Deaths	3 (13%)

^a^ Median (interquartile range); ^b^ two patients refused control examination; ^c^ data for one patient were not available; GDS, Guillain–Barré syndrome disability scale; MRCSS, Medical Research Council sum score.

**Table 2:** Clinical characteristics of GBS patients with type 2 diabetes.

Clinical Characteristics	Metformin Therapy		*p* Value
	yes (*n* =32)	no (*n* = 17)	
sex (male/female)	17/15	14/3	0.063 ^b^
age (years)	65 (57–68) ^a^	68 (61–72.5) ^a^	0.066 ^c^
fasting glycemia (mmol/L)	8.5 (6.0–12.2) ^a^	9.3 (7.1–12.1) ^a^	0.690 ^c^
Previous infection	13 (40.6%)	7 (41.2%)	1.000 ^b^
–gastrointestinal	5 (15.6%)	5 (29.4%)	0.285 ^b^
–respiratory	8 (25%)	2 (11.8%)	0.459 ^b^
IVIG and/or PE therapy	20 (62.5%)	11 (64.7%)	1.000 ^b^
GBS variant			
–AIDP	11 (34.4%)	8 (47.1%)	0.539 ^b^
–AMAN or AMSAN	10 (31.2%)	3 (17.6%)	0.498 ^b^
–undefined	11 (34.4%)	6 (35.3%)	1.000 ^b^
Mild GBS ^d^ at nadir	9 (28.1%)	0 (0%)	0.049 *^b^
Mild GBS ^d^ at discharge	21 (65.6%)	4 (23.5%)	0.007 *^b^

^a^ Median (interquartile range); ^b^ Fisher’s exact test; ^c^ Mann–Whitney U test (two-tailed); ^d^ GBS disability score ≤ 3; IVIG, intravenous immunoglobulins; PE, plasma exchange; AIDP, acute inflammatory demyelinating polyneuropathy; AMAN, acute motor axonal neuropathy; AMSAN, acute motor-sensory axonal neuropathy; * denotes a statistically significant difference.

**Table 3:** A predictive model for good GBS outcome in diabetic patients.

Variable	B	S.E.	Wald	df	*p*	OR	95% CI
Age	−0.033	0.046	0.531	1	0.466	0.967	0.885–1.058
Prior GIT infection	−2.600	1.154	5.078	1	0.024 *	0.074	0.008–0.713
Metformin therapy	1.729	0.747	5.367	1	0.021 *	5.637	1.305–24.352

B, regression coefficient; S.E., standard error of B; Wald, Wald test value; df, degrees of freedom; *p*, the significance of the Wald test (* denotes a statistically significant predictor); OR, odds ratio; CI, confidence interval.

## Data Availability

The data presented in this study are available in the article and Supplementary Materials.

## Supplementary Materials

The following supporting information can be downloaded at: https://www.mdpi.com/article/10.3390/cells11182897/s1, Figure S1: Original immunoblots of phospho/total AMPK and phospho/total LKB1; Figure S2: Original immunoblots of phospho/total Raptor and LC3-I/II; Figure S3: Original immunoblots of ATG5, beclin-1, and SQSTM1; Figure S4: Original immunoblots of phospho-4EBP1, phospho-ERK, and phospho-GSK3β; Figure S5: Original immunoblots of phospho-AKT and phospho-p38 MAPK; Figure S6: Original immunoblots of NBR1; Figure S7: Association between GBS severity and sex/age or prior infection; Figure S8: Expression of autophagy genes in PBMCs of GBS patients and control subjects; Figure S9: Correlation between AMPK, ERK, and PKC activation in PBMCs of GBS patients; Figure S10: Correlation between leukocyte AMPK signaling and GBS patients’ age; Figure S11: Correlation between leukocyte AMPK signaling and GBS severity; Figure S12: Correlation between autophagy markers/regulators and GDS at admission; Figure S13: Association between leukocyte autophagy markers/regulators and GBS outcome; Table S1: Addition of sex and fasting glycemia to a predictive model for good GBS outcome in diabetic patients.
