# Supplementary material for: Downregulation of LKB1/AMPK Signaling in Blood Mononuclear Cells Is Associated with the Severity of Guillain–Barre Syndrome
Source: Cells. 2022 Sep 16;11(18):2897. doi: 10.3390/cells11182897 (PMC9496801; doi:10.3390/cells11182897)
Supplement: Supplementary file 1 [file cells-11-02897-s001.zip › cells-1749926-supplementary.pdf]

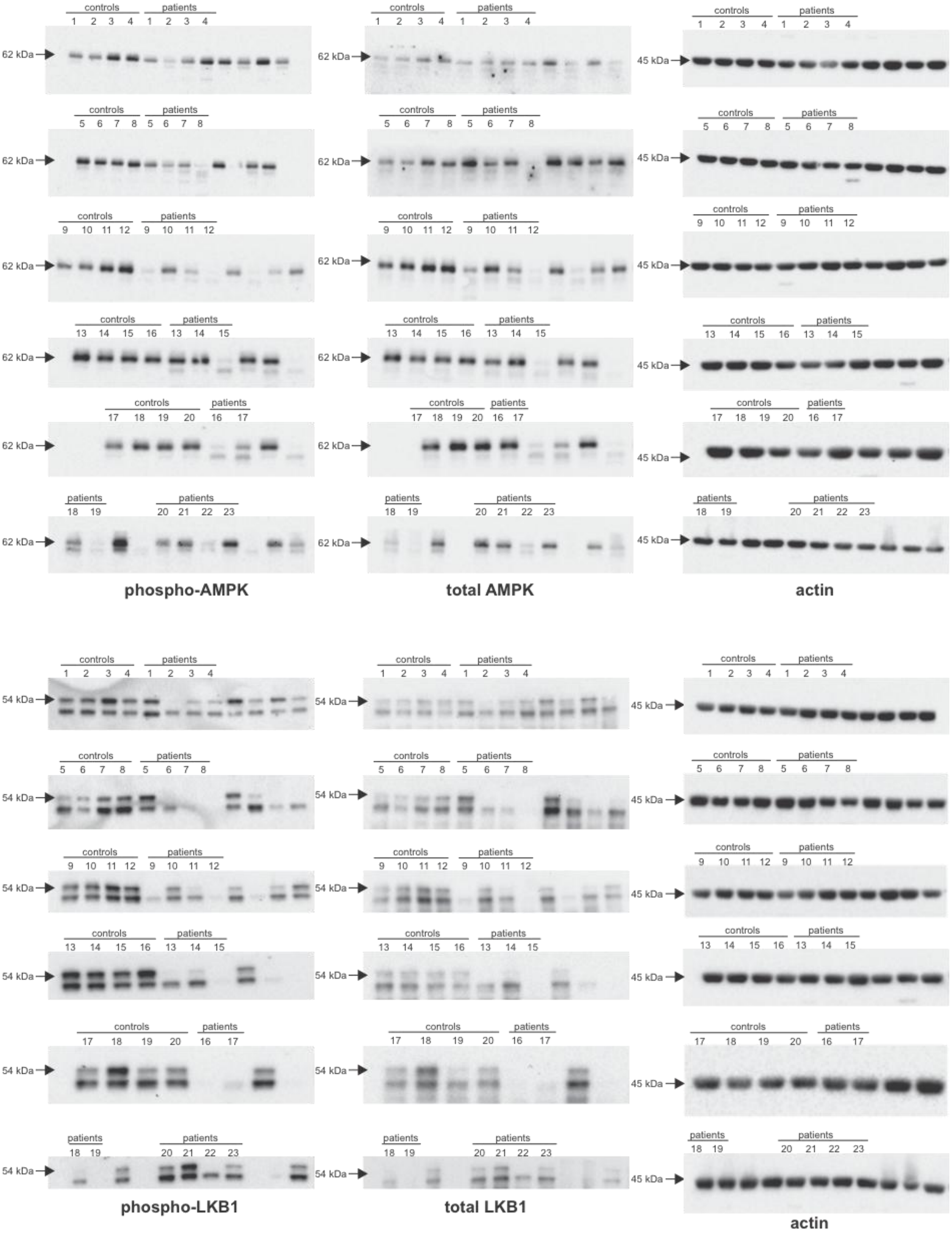

Figure S1. Original immunoblots of phospho/total AMPK and phospho/total LKB1.

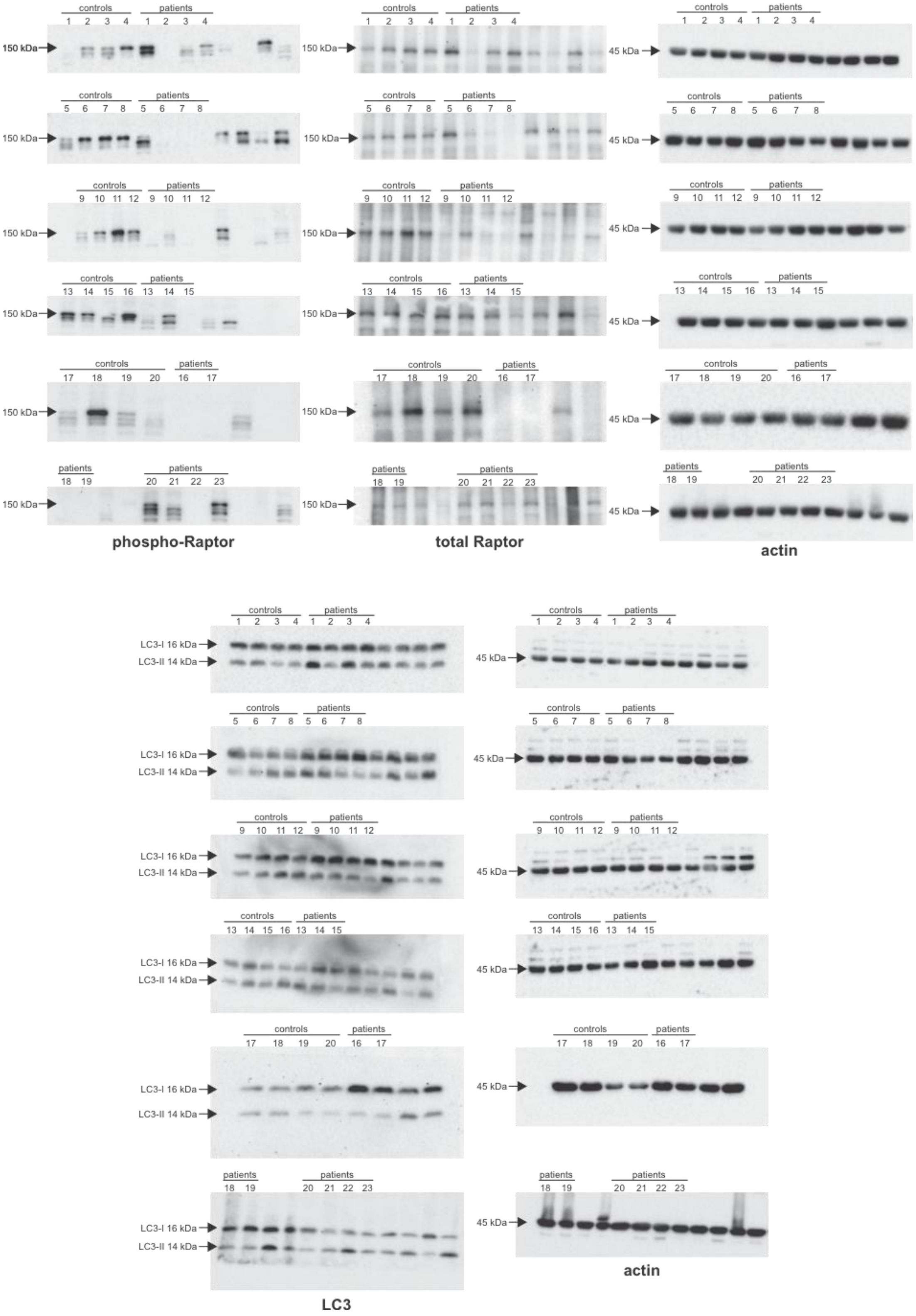

Figure S2. Original immunoblots of phospho/total Raptor and LC3-I/II.

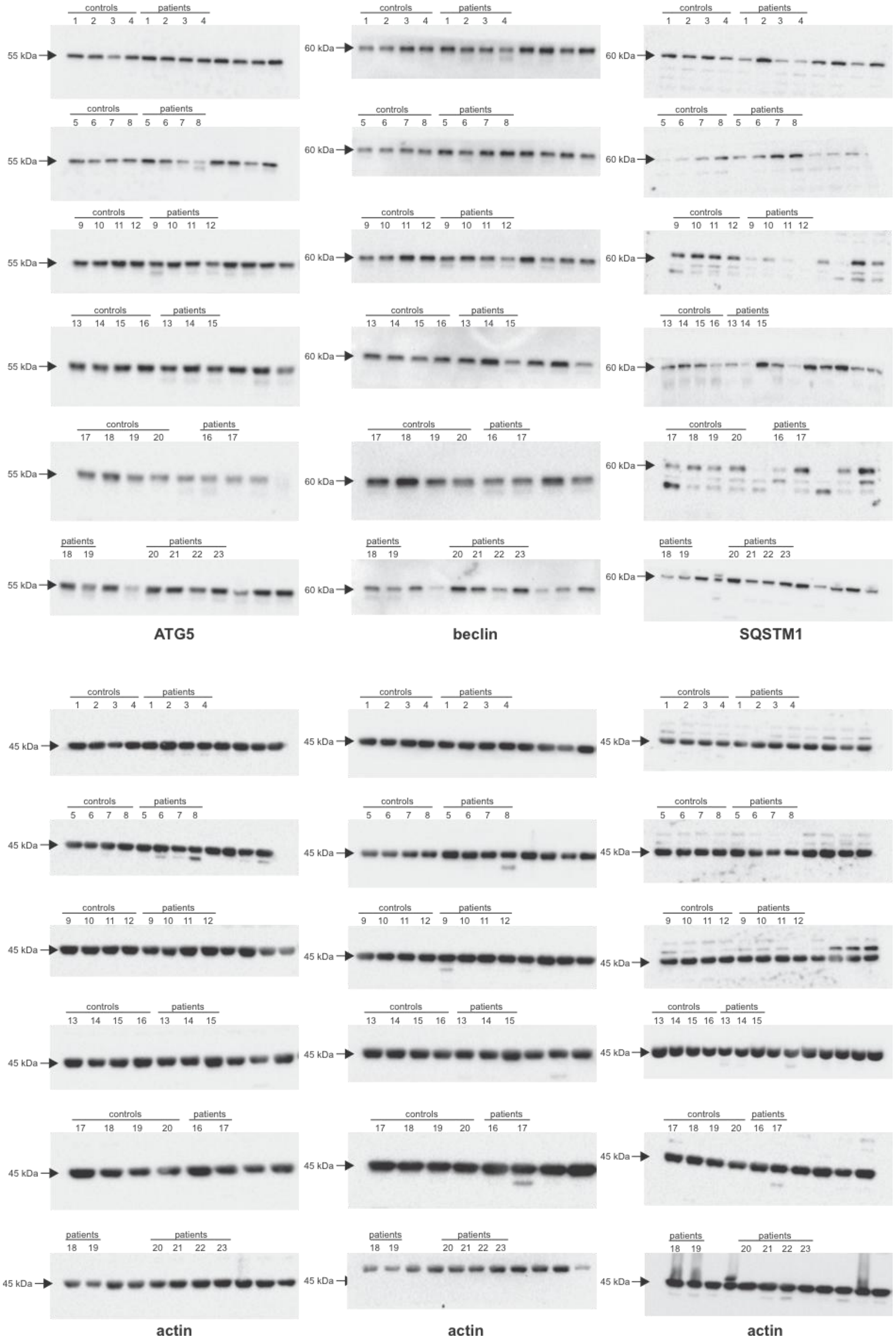

Figure S3. Original immunoblots of ATG5, beclin-1, and SQSTM1.

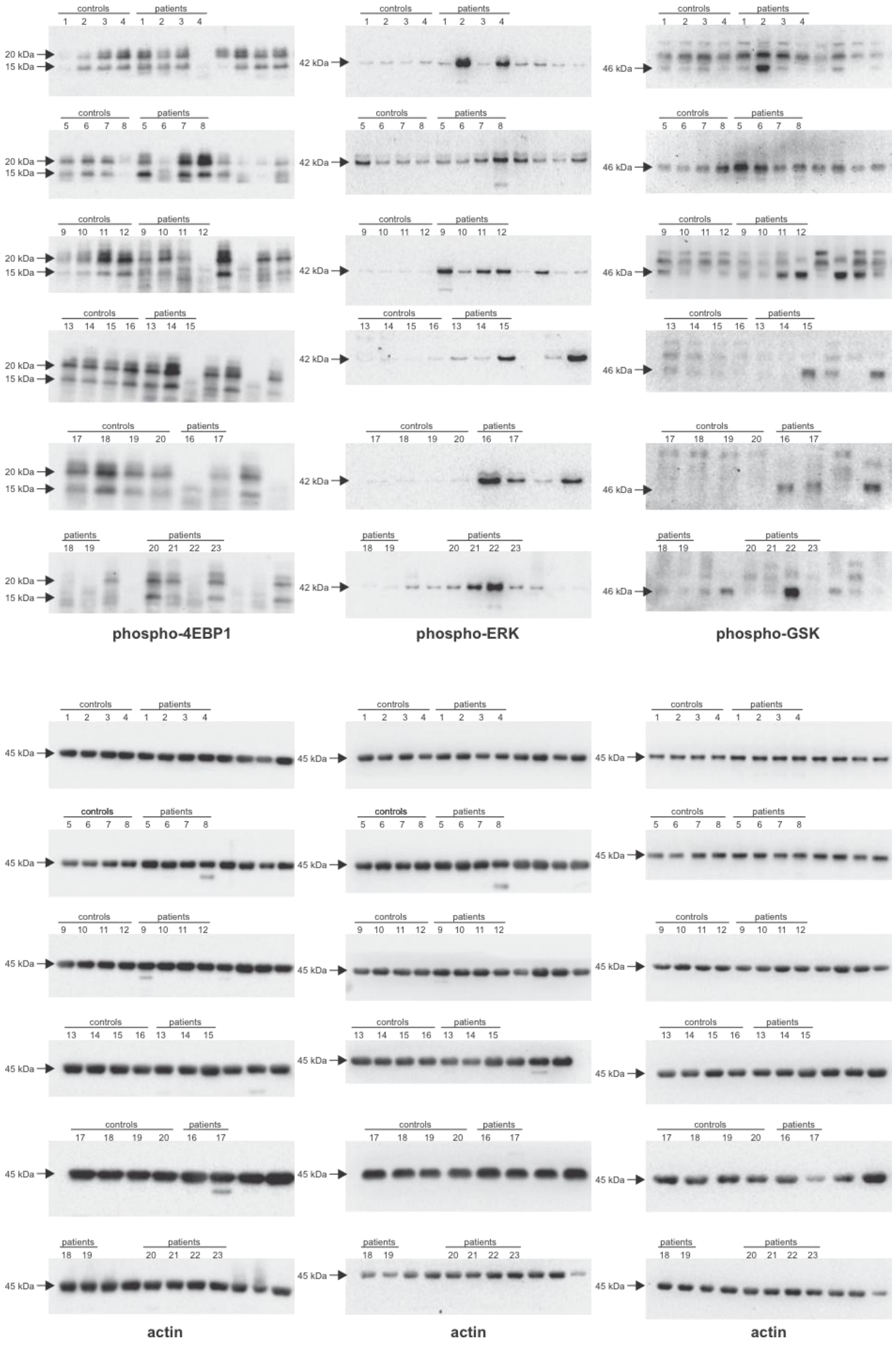

Figure S4. Original immunoblots of phospho-4EBP1, phospho-ERK, and phospho-GSK3β.

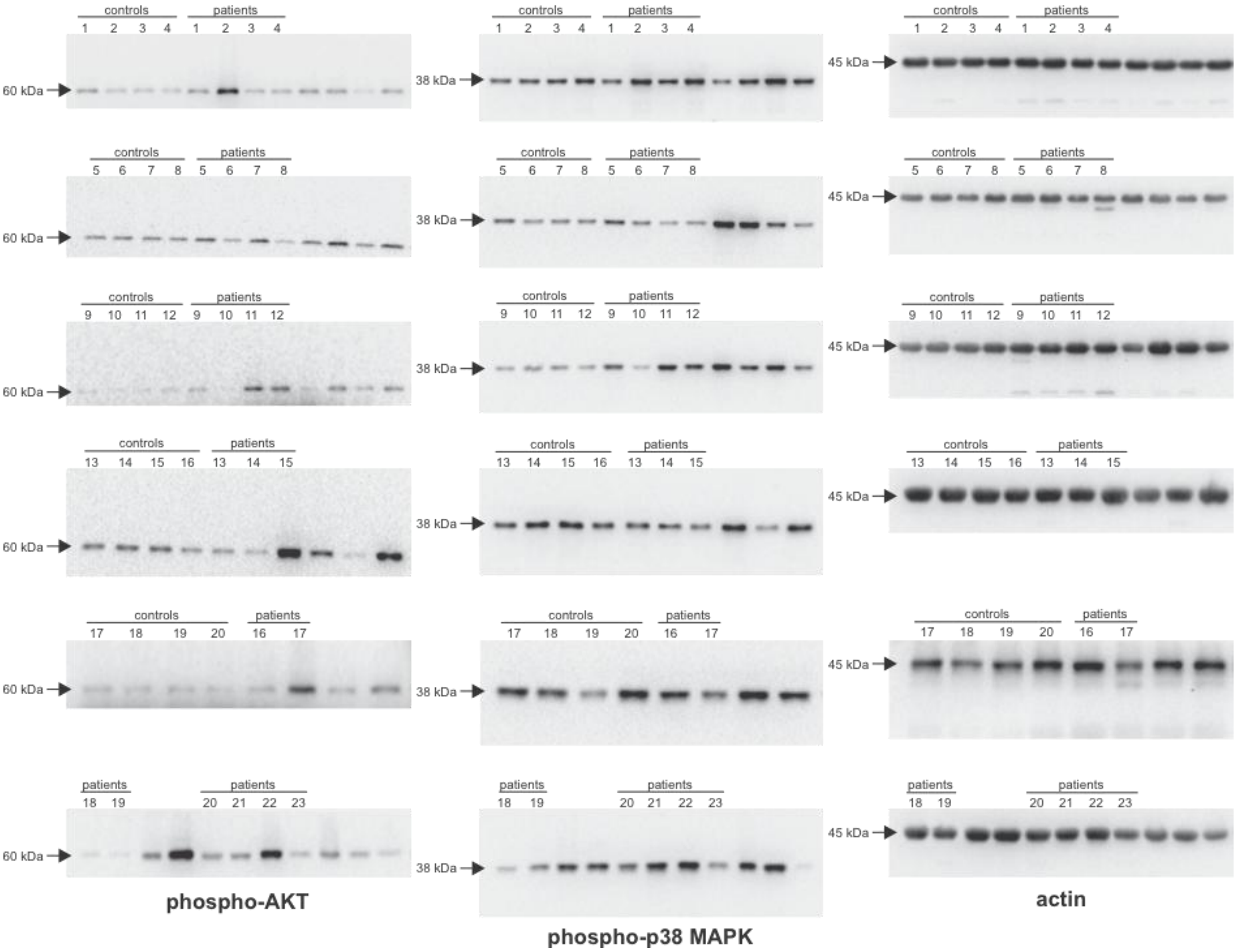

Figure S5. Original immunoblots of phospho-AKT and phospho-p38 MAPK.

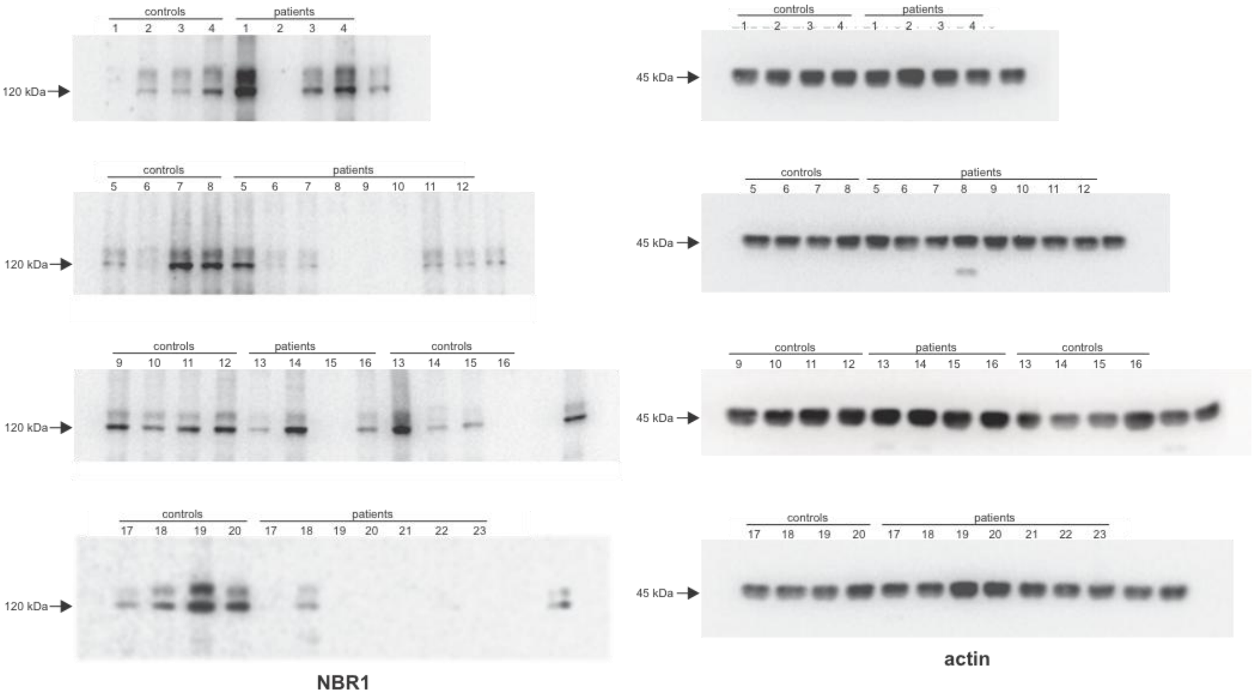

Figure S6. Original immunoblots of NBR1.

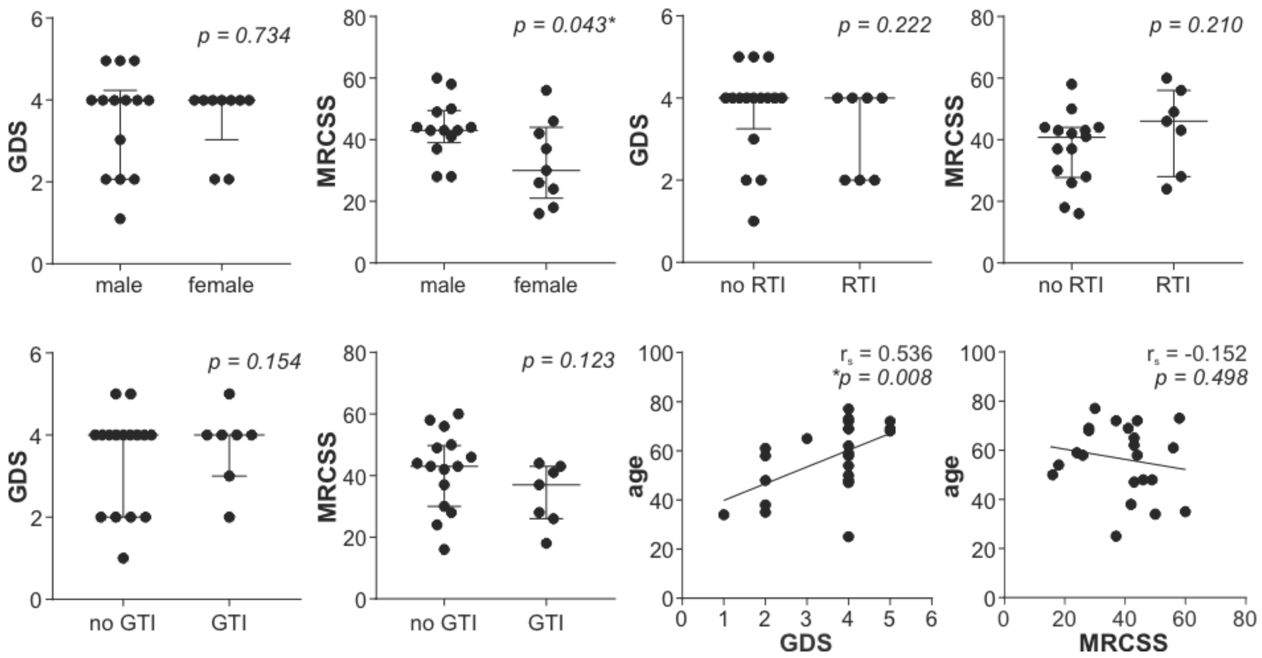

Figure S7. Association between GBS severity and sex/age or prior infection. Association between GDS or MRCSS and sex, prior infection, or age of GBS patients (n = 23) was assessed by the two-tailed Mann-Whitney U test (sex, prior infection) or Spearman's rank order test (age). GTI - gastrointestinal tract infection; RTI - respiratory tract infection; r_s - Spearman’s correlation coefficient; * denotes a statistically significant difference or correlation.

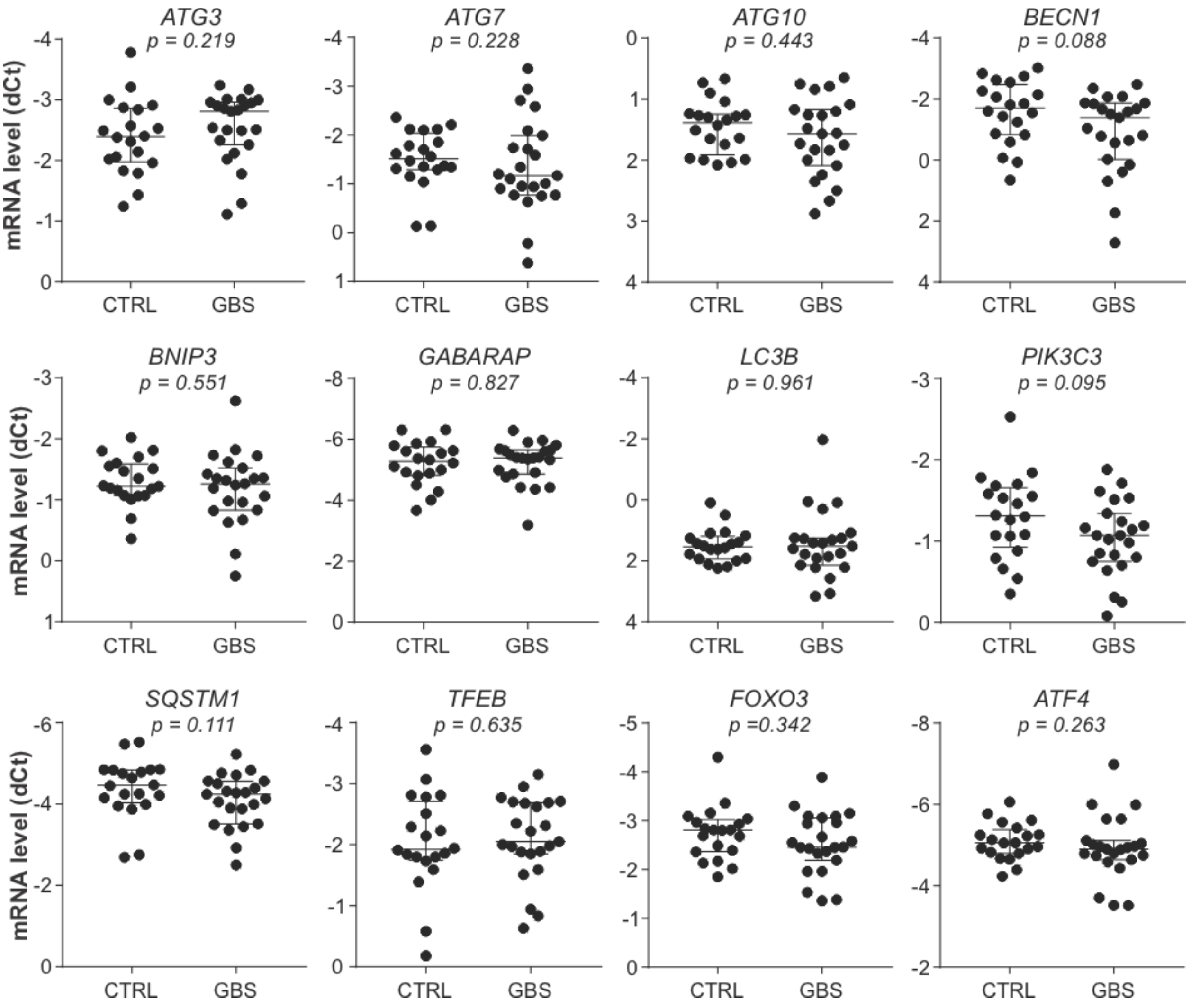

Figure S8. Expression of autophagy genes in PBMC of GBS patients and control subjects. Relative gene expression of target genes in PBMC of GBS patients (n = 23) and healthy control subjects (CTRL; n = 20) was determined by real-time RT-qPCR and presented as dCt values (lines represent median and interquartile range; p values were calculated by two-tailed Mann-Whitney U test).

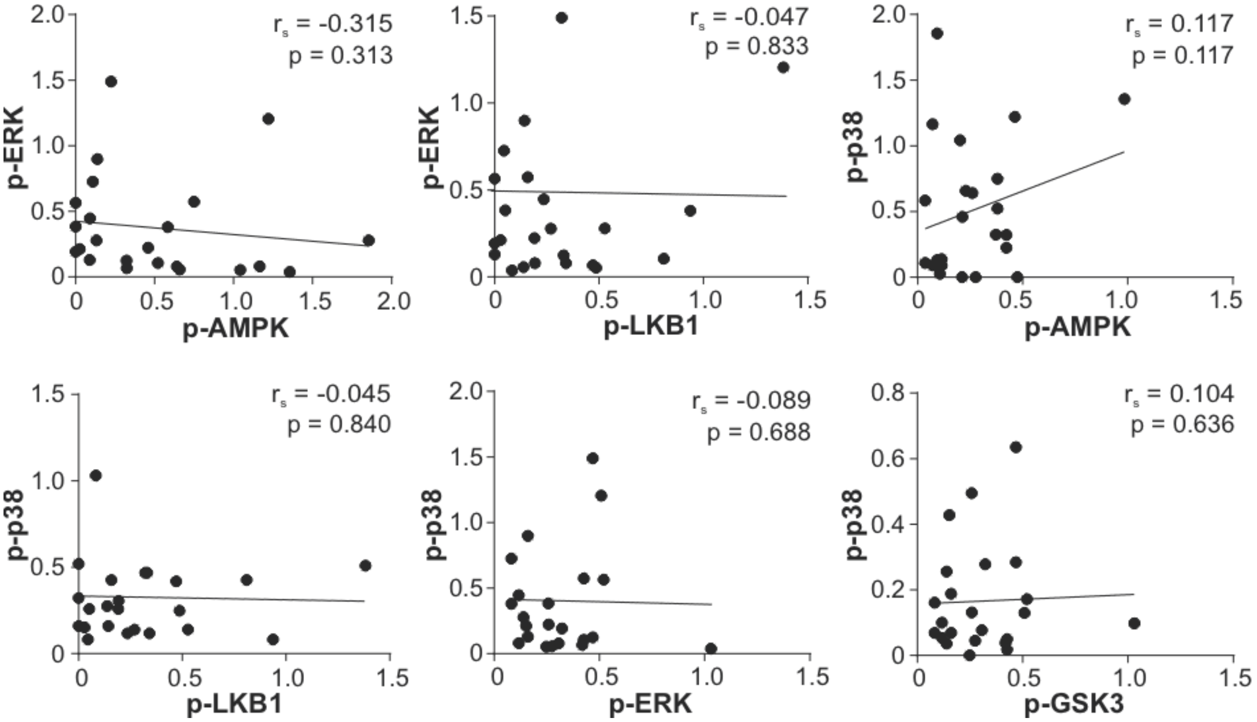

Figure S9. Correlation between AMPK, ERK, and PKC activation in PBMC of GBS patients. The levels of phosphorylated forms of AMPK, ERK, and PKC substrate GSK3β in PBMC of GBS patients (n = 23) were determined by immunoblotting, and the correlation plots are shown (r_s - Spearman’s correlation coefficient; p levels were calculated by Spearman's rank order test).

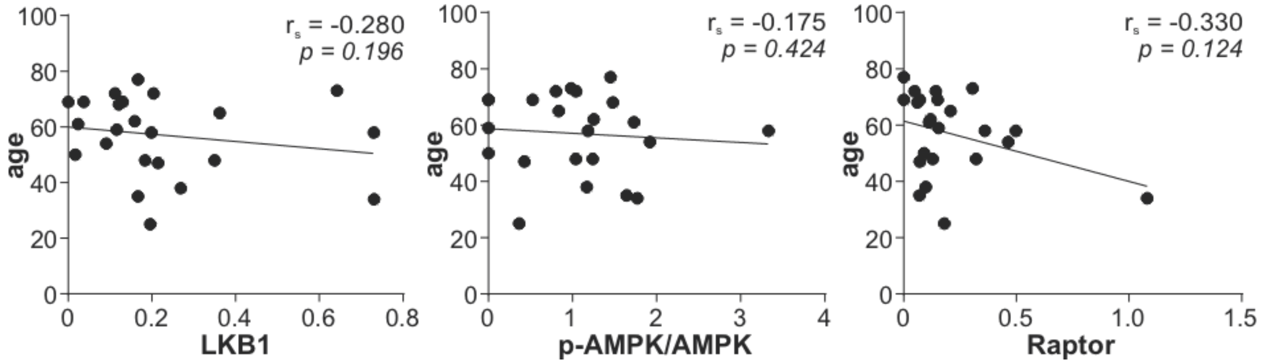

Figure S10. Correlation between leukocyte AMPK signaling and GBS patients' age. The levels of total LKB1, phospho/total AMPK, and total Raptor in PBMC of GBS patients (n = 23) were determined by immunoblotting, and the correlation with age was assessed (r_s - Spearman's correlation coefficient; p levels were calculated by Spearman's rank order test).

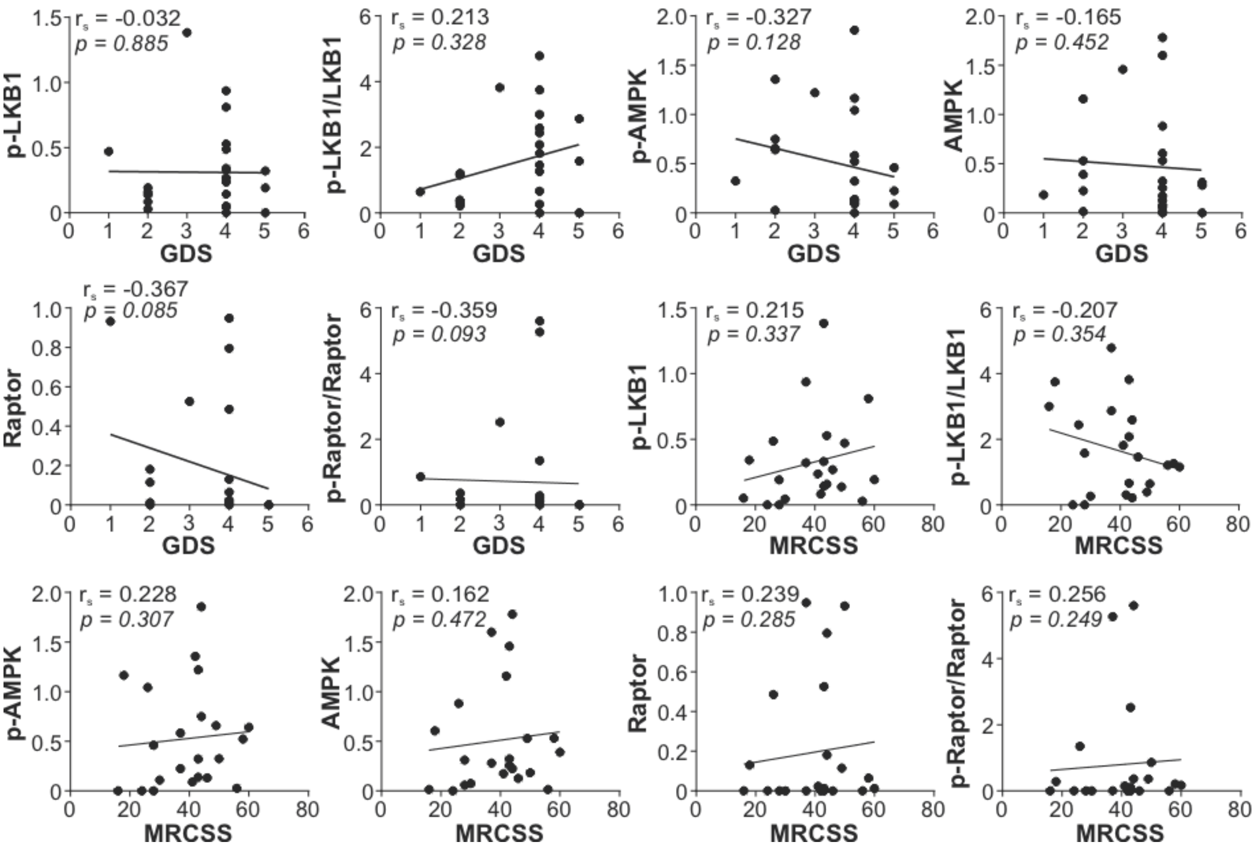

Figure S11. Correlation between leukocyte AMPK signaling and GBS severity. The levels of phospho-LKB1, phospho/total LKB1 ratio, phospho-AMPK, total AMPK, phospho-Raptor, and phospho/total Raptor ratio in PBMC of GBS patients were determined by immunoblotting, and the correlations with GDS (n = 23) and MRCSS (n = 22) were assessed (r_s - Spearman's correlation coefficient; p values were calculated by Spearman's rank order test).

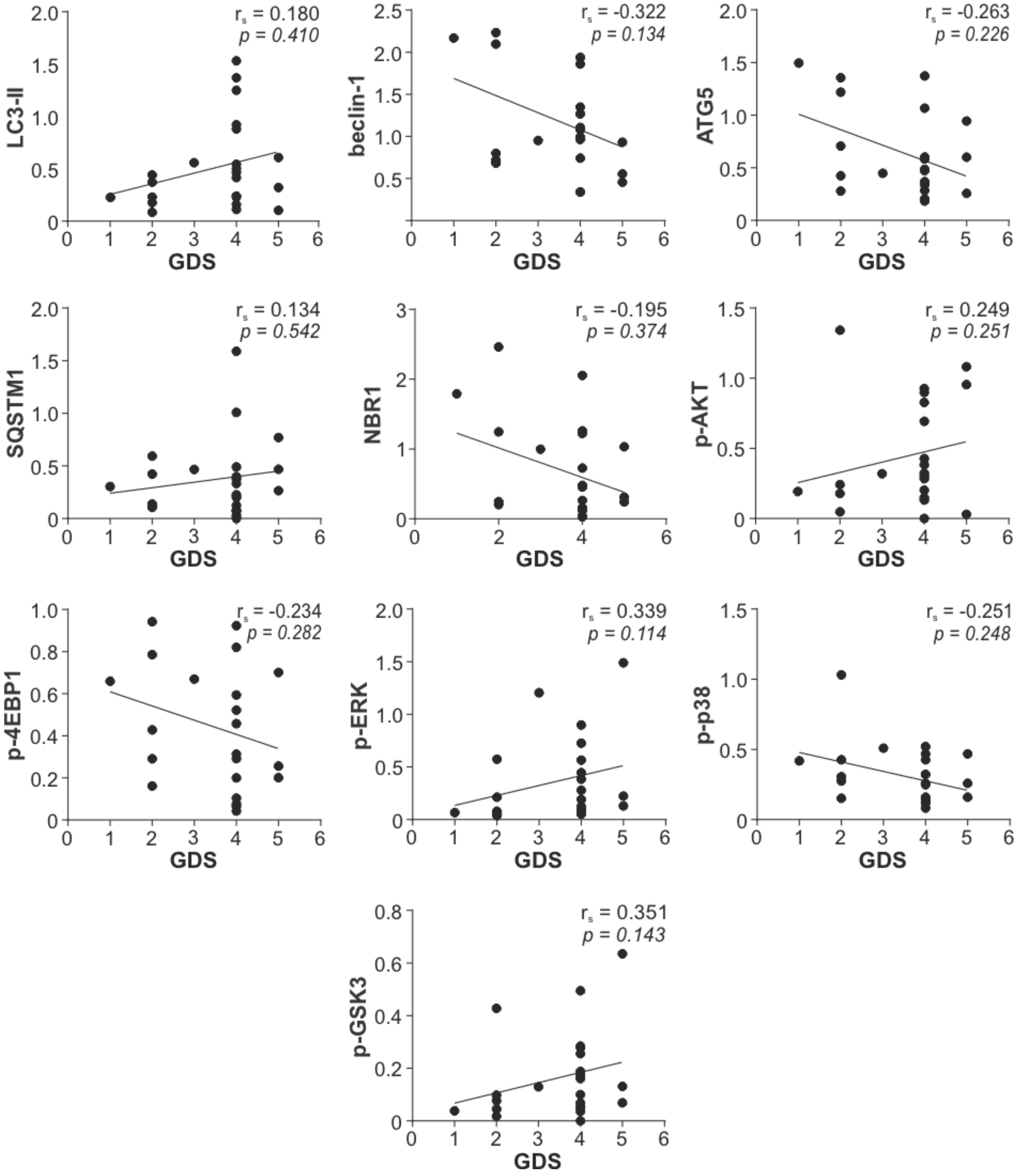

Figure S12. Correlation between autophagy markers/regulators and GDS at admission. The levels of LC3-II, beclin-1, ATG5, SQSTM1, NBR1, phospho-AKT, phospho-4EBP1, phospho-ERK, phospho-p38 MAPK, and pGSK3β in PBMC of GBS patients (n = 23) were assessed by immunoblotting, and the correlations with GDS were assessed (r_s - Spearman's correlation coefficient; p values were calculated by Spearman's rank order test).

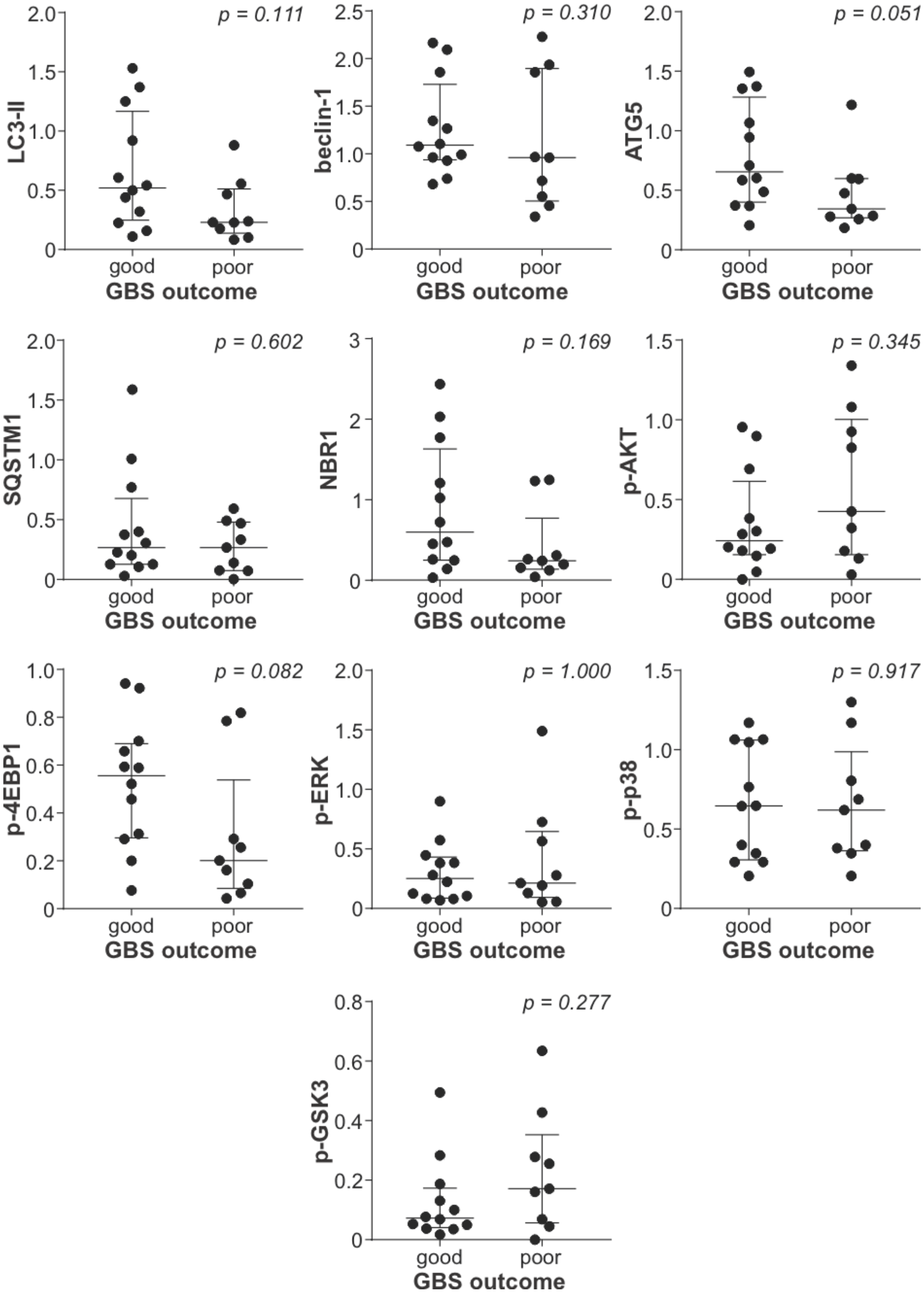

Figure S13. Association between leukocyte autophagy markers/regulators and GBS outcome. The levels of LC3-II, beclin-1, ATG5, SQSTM1, phospho-AKT, phospho-4EBP1, phospho-ERK, phospho-p38 MAPK, and pGSK3β in PBMC of GBS patients (n = 21) were assessed by immunoblotting and compared between patients with good (GDS ≤ 1, n = 12) and poor (GDS > 1, n = 9) disease outcome at 6 months (horizontal lines represent median and interquartile range; p values were calculated by two-tailed Mann-Whitney U test).

Table S1. Addition of sex and fasting glycemia to a predictive model for good GBS outcome in diabetic patients

Variable	B	S.E.	Wald	df	p	OR	95% CI
Age	-0.043	0.052	0.689	1	0.406	0.957	0.864-1.061
Prior GIT infection	-2.604	1.182	4.855	1	0.028*	0.074	0.007-0.750
Metformin therapy	1.666	0.766	4.734	1	0.030*	5.289	1.180-23.715
Sex (male)	-0.104	0.843	0.015	1	0.901	0.901	0.173-4.698
Fasting glycemia	-0.063	0.090	0.484	1	0.487	0.939	0.787-1.121

B, regression coefficient; S.E., standard error of B; Wald, Wald test value; df, degrees of freedom; p, significance of the Wald test (* denotes a statistically significant predictor); OR, odds ratio; CI, confidence interval for OR
